# Using a Failing Human Ventricular Cardiomyocyte Model to Re-Evaluate Ca^2+^ Cycling, Voltage Dependence, and Spark Characteristics

**DOI:** 10.3390/biom14111371

**Published:** 2024-10-28

**Authors:** Jerome Anthony E. Alvarez, Mohsin Saleet Jafri, Aman Ullah

**Affiliations:** 1 School of Systems Biology, George Mason University, Fairfax, VA 22030, USA; jalvar7@gmu.edu; 2 US Naval Research Laboratory, Center for Bio/Molecular Science and Engineering, Washington, DC 20375, USA; 3 Center for Biomedical Engineering and Technology, University of Maryland School of Medicine, Baltimore, MD 20201, USA

**Keywords:** calcium, heart failure, calcium sparks, ventricular myocyte, cardiac cell, ionic currents, RyR, LCC, Ca^2+^, computational modeling, heart, excitation contraction coupling

## Abstract

Previous studies have observed alterations in excitation–contraction (EC) coupling during end-stage heart failure that include action potential and calcium (Ca^2+^) transient prolongation and a reduction of the Ca^2+^ transient amplitude. Underlying these phenomena are the downregulation of potassium (K^+^) currents, downregulation of the sarcoplasmic reticulum Ca^2+^ ATPase (SERCA), increase Ca^2+^ sensitivity of the ryanodine receptor, and the upregulation of the sodium–calcium (Na^=^-Ca^2+^) exchanger. However, in human heart failure (HF), debate continues about the relative contributions of the changes in calcium handling vs. the changes in the membrane currents. To understand the consequences of the above changes, they are incorporated into a computational human ventricular myocyte HF model that can explore the contributions of the spontaneous Ca^2+^ release from the sarcoplasmic reticulum (SR). The reduction of transient outward K^+^ current (I_to_) is the main membrane current contributor to the decrease in RyR2 open probability and L-type calcium channel (LCC) density which emphasizes its importance to phase 1 of the action potential (AP) shape and duration (APD). During current-clamp conditions, RyR2 hyperphosphorylation exhibits the least amount of Ca^2+^ release from the SR into the cytosol and SR Ca^2+^ fractional release during a dynamic slow–rapid–slow (0.5–2.5–0.5 Hz) pacing, but it displays the most abundant and more lasting Ca^2+^ sparks two-fold longer than a normal cell. On the other hand, under voltage-clamp conditions, HF by decreased SERCA and upregulated I_NCX_ show the least SR Ca^2+^ uptake and EC coupling gain, as compared to HF by hyperphosphorylated RyR2s. Overall, this study demonstrates that the (a) combined effect of SERCA and NCX, and the (b) RyR2 dysfunction, along with the downregulation of the cardiomyocyte’s potassium currents, could substantially contribute to Ca^2+^ mishandling at the spark level that leads to heart failure.

## 1. Introduction

The complex and coordinated mechanism of calcium-induced calcium release (CICR) in ventricular cardiomyocytes has been rigorously studied to better understand the processes behind excitation–contraction coupling (ECC) in cardiac cells [1]. In a healthy human heart, the process of contraction starts with the opening of the voltage-dependent calcium (Ca^2+^) channels also called dihydropyridine receptors (DHPRs) or the L-type calcium channel (I_LCC_). The opening of DHPRs with depolarization, however, is insufficient to induce a full contraction. This small influx of Ca^2+^ from the I_LCC_ induces a Ca^2+^ release from the sarcoplasmic reticulum (SR) through the ryanodine receptors (RyR2s) in a process termed calcium-induced calcium release (CICR). This depolarizing Ca^2+^ influx in CICR activates the RyR2s which release Ca^2+^ from the sarcoplasmic reticulum into the myoplasm ([Ca^2+^]_myo_) which results in a myocyte contraction through the shortening of the sarcomere. In the repolarization process, SR Ca^2+^ levels are then re-sequestered by the SR Ca^2+^–ATPase (SERCA) from the myoplasm. Excess Ca^2+^ is further extruded from the myoplasm by the sodium–calcium exchanger (Na^+^–Ca^2+^; I_NCX_) which renders the cardiac cells into a “relaxed” state.

On the other hand, asynchronous or erratic Ca^2+^ cycling is a prominent characteristic of a failing human heart. Impaired SERCA function and enhanced I_NCX_ activity have been proposed as causes of reduced SR Ca^2+^ load notable in cases of heart failure (HF) [2]. The dysfunction of Ca^2+^ dynamics during heart failure leads to early and/or delayed afterdepolarizations (EADs and/or DADs) initiates ventricular arrhythmia [3,4]. These phenomena exhibit secondary depolarization occurring before or after a complete action potential (AP) repolarization. These arrhythmias can lead to ventricular tachycardia which often serves as an immediate precursor to ventricular fibrillation which has been identified as a major cause of sudden cardiac death [5]. While these observations are generally accepted in recent studies, further understanding of excitation–contraction coupling dynamics during these conditions is needed.

### Impaired SERCA vs. Hyperphosphorylated RyR2

The mechanisms of heart failure continue to be a subject of discussion and disagreement in the literature. One perspective, as previously mentioned, is the combined effect of decreased SERCA and increased I_NCX_ activities. Another is the diastolic SR Ca^2+^ leak via calstabin2-depleted “leaky” RyR2 channels caused by phosphorylation [2,6]. The latter has been increasingly recognized as an important contributor to altered Ca^2+^ handling in failing hearts. Relatedly, the amplitude and rate of decay of both contraction and the underlying systolic Ca^2+^ transient ([Ca^2+^]_i_) are reduced [6]. A noteworthy study by Jiang et al. states that RyR2 activities in both failing and healthy human and canine hearts appear structurally and functionally normal, meaning that their differences in RyR open probabilities are not statistically significant [7]. This is also supported by the findings of Stange et al. wherein single site RyR2 phosphorylation does not substantially change between wild-type and mutant RyR2s [8]. Moreover, studies by Lehnart et al. and Marx et al. suggest that hyperphosphorylated RyR2 channels are associated with cardiac dysfunction and arrhythmias which overall contributes to defective Ca^2+^ regulation in failing hearts [9,10]. Ultimately, the mechanisms behind the reduction of SR Ca^2+^ content and impaired cardiac contractility can be traced from the following: (a) the impaired SERCA activity accompanied by increased I_NCX_, (b) an increased Ca^2+^–RyR2 sensitivity or hyperphosphorylated RyR2 channels, or (c) both conspiring to deplete the SR Ca^2+^ [2,11]. Ultimately, the mechanisms behind the reduction of SR Ca^2+^ content and impaired cardiac contractility can be traced from: (a) the impaired SERCA activity accompanied by increased I_NCX_, (b) an increased Ca^2+^–RyR2 sensitivity or hyperphosphorylated RyR2 channels, or (c) both conspiring to deplete the SR Ca^2+^ [2,11]. A reevaluation of these factors is emphasized and suggested by the works of Eisner and Trafford [6].

Although multiple observations have been reported in targeting principal mechanisms of heart failure, further investigation at the level of the Ca^2+^ spark is needed. This study presents an overall comparison of Ca^2+^ dynamics and sparks between SERCA/NCX and RyR2 phosphorylation in failing human ventricular cardiomyocytes, along with its attributable changes in AP morphology and EC coupling gain. The force–frequency relationship between healthy and failing hearts through predicted steady-state force and SR fractional release are also further explored in this research.

## 2. Materials and Methods

### 2.1. Model

Simulations were performed using a previously published stochastic human ventricular cardiomyocyte model [12]. The cardiomyocyte’s calcium-release units (CRUs) in the cardiac dyad consist of L-type calcium channels (LCCs) in t-tubules which are co-localized with ryanodine receptors (RyR2s; type 2 in cardiac cells) in a junctional SR (JSR) membrane. The 20,000 CRUs in this model are composed of 9 individual LCCs and 49 RyRs that function as couplons and they are intricately connected through the complex organization of the network SR (NSR) which stores the main intracellular calcium in cardiac muscle cells. Moreover, the excitation–contraction coupling scheme incorporates 3-state RyR and 6-state L-type Ca^2+^ channel mode switching that adapt to various calcium dynamics and action potential morphology consistent with experimental findings (see [12] for further details).

### 2.2. Statistical Analysis

Analysis of Variance (ANOVA) and pairwise comparisons using Student’s paired *t*-Tests as specified were conducted using Microsoft Excel (Redmond, WA, USA). Figures were generated using R Software’s (version 2023.06.1, Vienna, Austria) base, stats, and ggplot2 packages, MATLAB 2016a (Natick MA), and IDL^®^ Software (Ballsbridge, London)—L3HarrisGeospatial. Statistical tests were considered statistically significant at *p* < 0.05.

### 2.3. Pacing Protocol

Simulations and analyses were performed at 1 Hz for 20 s to achieve steady state unless specified otherwise. Dynamic pacing was conducted using slow–rapid–slow pacing at 0.5 Hz–2.5 Hz–0.5 Hz for determining the force–frequency relationship. Voltage-clamp experiments were conducted in both control and heart-failure conditions: measurements of Ca^2+^ dynamics were activated by a series of 0.2 s depolarizing steps (V_Test_) from −40 to +30 mV in increments of 5 mV.

### 2.4. Force–Frequency Relationship and SR Ca^2+^ Fractional Release

Sun and Irving [13] described the molecular basis of the force–calcium relation in the heart muscle. Changes in [Ca^2+^]_i_ can directly affect cardiac contractility which helps distinguishing factors in a defective cardiac cell. The co-operative mechanism of Ca^2+^ dependence in force generation was shown to be an intrinsic property of the thin filaments (i.e., actin, tropomyosin, troponin) during contraction. This Ca^2+^ dependence of isometric force generation was experimentally observed using demembranated ventricular trabeculae by Sun et al. [14] The predicted force using intracellular Ca^2+^ is described by the Hill equation:

(1) $$force=\frac{1}{1+10^{n_{H}(pCa-{pCa}_{50})}}$$

where $n_{H}$ (ranging 3 to 4) is the Hill coefficient, pCa represents peak systolic [Ca^2+^]_myo_ in log scale ($pCa=-{log}_{10}{\left[{Ca}^{2+}\right]}_{myo})$, and ${pCa}_{50}$ (ranging 5.5 to 6.0) corresponds to half-maximum force. For this study, $n_{H}=3$ and ${pCa}_{50}=6$ were used in computing the predicted force for each peak [Ca^2+^]_myo_ concentration.

On the other hand, the fraction of the SR Ca^2+^ content released at a twitch, known as “fractional SR Ca^2+^ release”, is an important parameter for assessing the efficiency of excitation–contraction coupling under physiological and pathophysiological conditions [15,16]. The global SR Ca^2+^ depletion amplitudes in both diastolic and systolic phases of the SR Ca^2+^ are recorded in this study, and the fractional release is computed by:

(2) $$Fractional\;Release=\frac{[{Ca}^{2+}{]}_{SR,diastolic}-[{Ca}^{2+}{]}_{SR,systolic}}{[{Ca}^{2+}{]}_{SR,diastolic}}$$

## 3. Results

### 3.1. Simulated Excitation–Contraction Coupling Changes in Heart Failure

**Figure 1:**
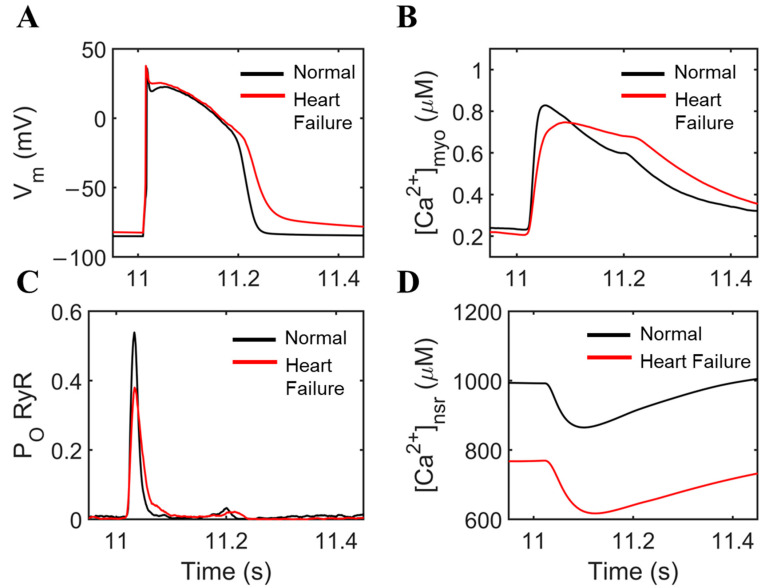
Comparisons of normal cardiac cell conditions vs. heart failure. To ensure steady state conditions, 20 s simulations at 1 Hz were performed. (**A**) APD prolongation with longer plateau phase due to influx of Ca^2+^. Early afterdepolarizations (EADs) were also observed through the oscillations of the membrane potential. (**B**) Ca^2+^ concentration displays an incomplete extrusion from the myoplasm. (**C**) RyR2 open probability is decreased during heart failure, and small openings were observed during EAD occurrence. (**D**) SR Ca^2+^ also displays incomplete recovery, mainly due to decreased SERCA activity and increased NCX expression.

Alterations in Ca^2+^ handling contribute to diminished contraction and relaxation in the event of heart failure (Figure 1). Ca^2+^ homeostasis is essential to a normal functioning heart; however, abnormalities in Ca^2+^ dynamics are key determinants in the development of arrhythmogenic disorders in the failing heart. In CICR, extracellular calcium entry into the cell through ion channels in the T-tubules leads to an increase in calcium concentration in the dyadic subspace. The model in this study examined how subspace [Ca^2+^]_i_ changes between control and heart-failure conditions by implementing previously observed changes in ion transport proteins [17,18,19]. The overall simulation settings are summarized in Table 1 simulate end-stage heart failure. The K^+^ currents (I_to_, I_K1_, I_Kr_, and I_Ks_) were downregulated in all cases of failing hearts in this study, specifically ventricular samples from explanted hearts from end-stage HF patients undergoing transplantation and similar cases [20,21,22]. NCX protein expression was increased by 100% and SR Ca^2+^ ATPase (SERCA) protein was decreased by 30% [19,23]; and RyR2 Ca^2+^ sensitivity was increased by 50% to mimic increased activity from chronic hyperphosphorylation [9,24,25,26,27]. Overall, these ionic effects lead to impaired cellular functions observed in failing heart cells.

**Figure A1:**
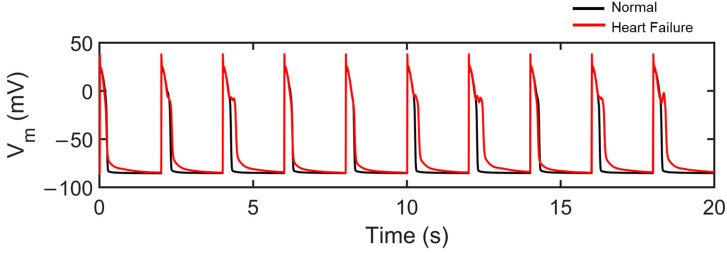
Early afterdepolarizations. Remodeled HF is paced at 0.5 Hz to induce EADs. Five EADs were observed throughout the 20 s simulated HF.

Simulations at 1 Hz pacing displayed APD prolongation and a loss of the distinctive “notch” in the action potential shape during HF similar to the experiment (Figure 1A). Multiple EADs were also observed during a 20 s simulation of heart failure under slow 0.5 Hz pacing (Appendix A Figure A1) which was also demonstrated by Li and colleagues in canine hearts [31] where EADs occur more than 40% on average during a slow 0.5 Hz frequency. On the other hand, myoplasmic Ca^2+^ concentration displays a slower decay due to attenuated sequestration back to the SR due to reduced SERCA function (Figure 1B); a small “notch” can also be observed in HF (red) and this phenomenon was demonstrated in experimental studies [32,33,34]. The RyR open probability shows decrease peak and more dispersed activation late in the AP under HF conditions (Figure 1C) which is further discussed in the next section. Lastly, because SR Ca^2+^ influx is directly influenced by SERCA in transporting Ca^2+^ from the myoplasm—the SERCA is impaired in this case—overall levels of SR Ca^2+^ concentration are decreased (Figure 1D), which can account for the reduced RyR open probability in spite of the increased Ca^2+^ sensitivity because of RyR phosphorylation. Furthermore, the decreased SR Ca^2+^ content also reduces the driving force for Ca^2+^, contributing to the reduced amplitude of the Ca^2+^ transient (Figure 1B). These results are consistent with the early work of Vermeulen and colleagues [35,36] using human ventricular trabeculae from patients with terminal heart failure and further simulation studies from Priebe and Beuckelmann [37].

### 3.2. Contributions of K^+^ Currents to Heart-Failure Phenotype

Several experimental studies have shown varying degrees of protein expression of the repolarizing K^+^ currents in end-stage, diseased, and failing human ventricular cells [20,22,28]. Specifically, the inward rectifying (I_K1_), transient outward (I_to_), and the slow delayed rectifier (I_Ks_) K^+^ currents are downregulated in all cases of cells exhibiting abnormal histology. The downregulation of the rapid delayed rectifier (I_Kr_) K^+^ current in failing human ventricular myocytes remains controversial in the literature due to statistically non-significant results, but nevertheless, a shift in the hERG1a:hERG1b isoform stoichiometry at the protein level was observed [22] (further analysis of this case is explained in Section 4. Discussion). This study therefore includes its potentially arrhythmogenic contribution to APD prolongation.

**Figure 2:**
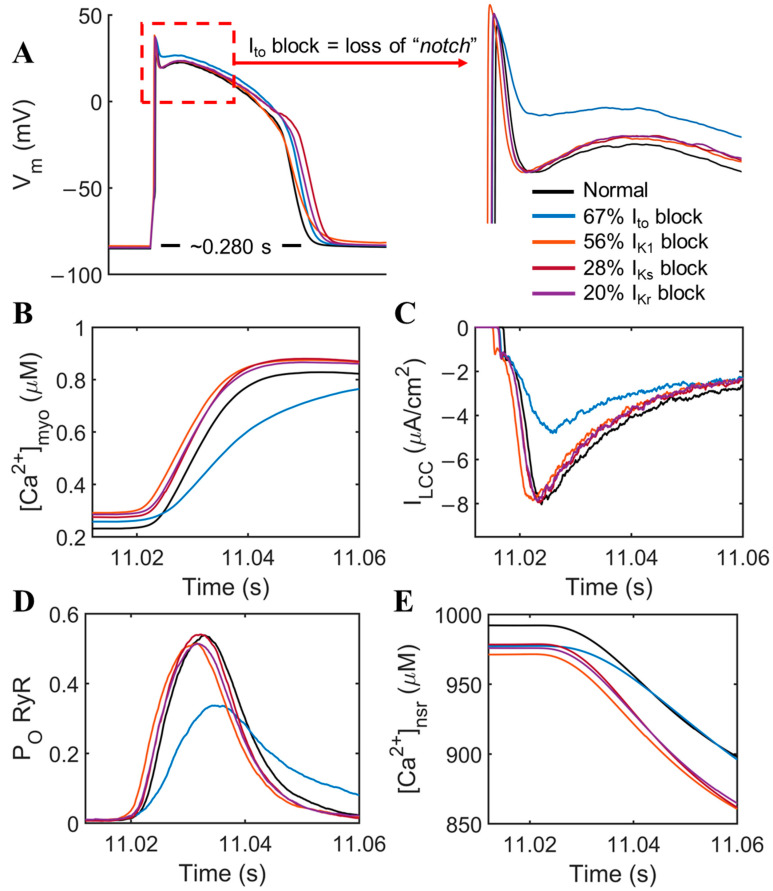
Prominent effects of I_to_ among other K^+^ currents. (**A**) APD prolongation from the downregulated repolarizing K^+^ currents present in failing human cardiomyocytes with decreased I_to_ (blue) showing a loss of the distinctive “notch” in the action potential shape. (**B**) Roughly two-fold increase in time-to-peak Ca^2+^ transient from 0.02 to 0.04 s duration was observed. (**C**) I_LCC_ peak density also decreased roughly 30–35%, respectively, by the effects of I_to_ alone. (**D**) RyR open probability is reduced ~20–25% and (**E**) delayed kinetics of Ca^2+^ release from the SR by I_to_.

The differential expressions from the reported voltage-dependent K^+^ currents in heart failure could provide defining characteristics in the action potential and calcium dynamic profiles in cardiac cells. From the data in Table 1, each respective downregulation of the K^+^ currents was factored into the reduction of conductance without the effects of SERCA, I_NCX_, and RyR modulations (Figure 2A). All K^+^ current blocks can potentially prolong the APD, but I_to_ shows a prominent effect on the “notch”, which is a distinctive feature of the human AP. This effect is expected because I_to_ is responsible for the repolarizing current in phase 1 of the action potential, but its other consequences are also observed in other channels and cellular Ca^2+^ concentrations.

One of the known arrhythmia mechanisms in cardiac hypertrophy and heart failure is a slowed Ca^2+^ transient [38,39]. However, there is little information on the possible effects of K^+^ currents in the prolonged time-to-peak of intracellular Ca^2+^ from the SR. Hence, we tested each of the K^+^ current modifications as previously stated in Table 1 simulation settings. We observed that I_to_ shows a notable effect on slowed time-to-peak Ca^2+^ transient from ~0.02 to ~0.04 s (Figure 2B). I_LCC_ peak density also decreased approximately 30–35% (Figure 2C) from the effects of downregulated I_to_ alone. This occurs because the loss of the notch (increased membrane potential) reduces the driving force for Ca^2+^. Moreover, the reduction in I_LCC_ reduces the number of dyads activated and thus reduces RyR2 open probability by ~20–25% (Figure 2D). This contributes to the delayed kinetics of SR Ca^2+^ release into the cytosol (Figure 2E) and occurs mainly through the reduction in I_to_ compared with other repolarizing currents.

**Figure 3:**
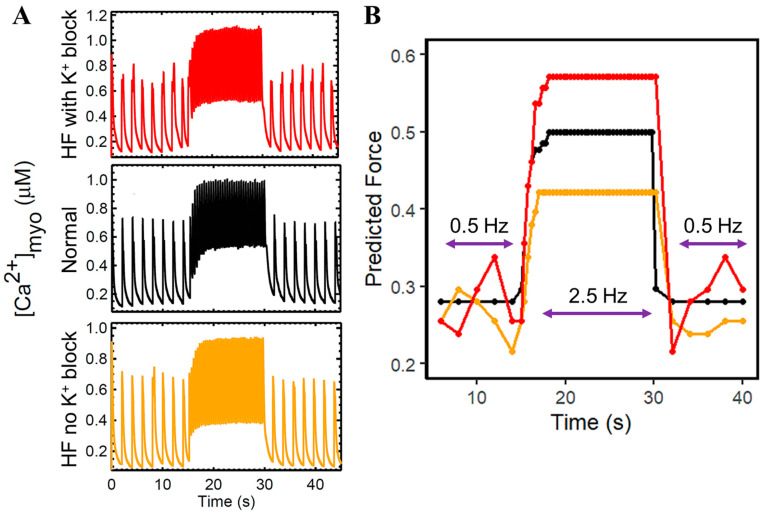
Dynamic slow–rapid–slow pacing (0.5–2.5–0.5 Hz) [Ca^2+^]_myo_ concentrations between normal, HF with K^+^ block (i.e., It_o_, I_K1_, I_Kr_, and I_Ks_), and HF without K^+^ block. To ensure steady state conditions, 45 s simulations were performed. (**A**) Erratic Ca^2+^ transient peaks were observed during 0.5 Hz pacing in failing hearts. HF with reduced K^+^ currents (red) slightly elevated the [Ca^2+^]_myo_ amplitude during high pacing (2.5 Hz) as compared to normal (black) and HF without K^+^ reductions (yellow). (**B**) Predicted force using the same color schemes. HF with reduced K^+^ currents (red) exhibit higher predicted force using peak systolic [Ca^2+^]_myo_ concentrations in rapid pacing.

Figure 3 explores force–interval relations. Because of the decreased repolarizing K^+^ currents shown in Figure 2B, the Ca^2+^ transient also exhibits a small increase in amplitude, with the exception of I_to_’s effects (in blue). The APD is generally prolonged due to these effects and more Ca^2+^ is transported inside the cell.

The next set of simulations uses the slow–rapid–slow pacing (0.5–2.5–0.5 Hz) protocol to explore excitation–contraction coupling potentiation during rapid pacing. In the context of Ca^2+^ dependence of isometric force generation using peak systolic [Ca^2+^]_myo_ (Equation (1)), dynamic slow–rapid–slow pacing (Figure 3) shows that the potentiation of the Ca^2+^ transient and force that occurs with rapid pacing is attenuated with the changes to RyR sensitivity, SERCA, and NCX (red) vs. normal healthy myocytes. The changes in the membrane K^+^ current attenuates the potentiation during the rapid pacing at 2.5 Hz. High pacing of 2.5 Hz was chosen as the peak frequency for this simulation, which was observed to reach the maximal developed force in humans from experimental recordings [40].

### 3.3. RyR2 Hyperphosphorylation vs. Impaired SERCA and Upregulated NCX

Competing hypotheses of key determinants in the altered Ca^2+^ cycling of failing hearts have long been debated. Maladaptive protein kinase hyperphosphorylation of RyR2 in failing hearts alters channel function, which may cause SR Ca^2+^ depletion and its diastolic release [2]. On the other hand, the widespread agreement in earlier studies suggests that a decreased SR Ca^2+^ content was due to a decrease in SERCA coupled with an increase in NCX, which decreases the capacity of the SR to accumulate calcium [41]. However, they suggest that the relative contributions of changes of SERCA and RyR2 to heart failure be reevaluated [6], which will was done in this study.

**Figure 4:**
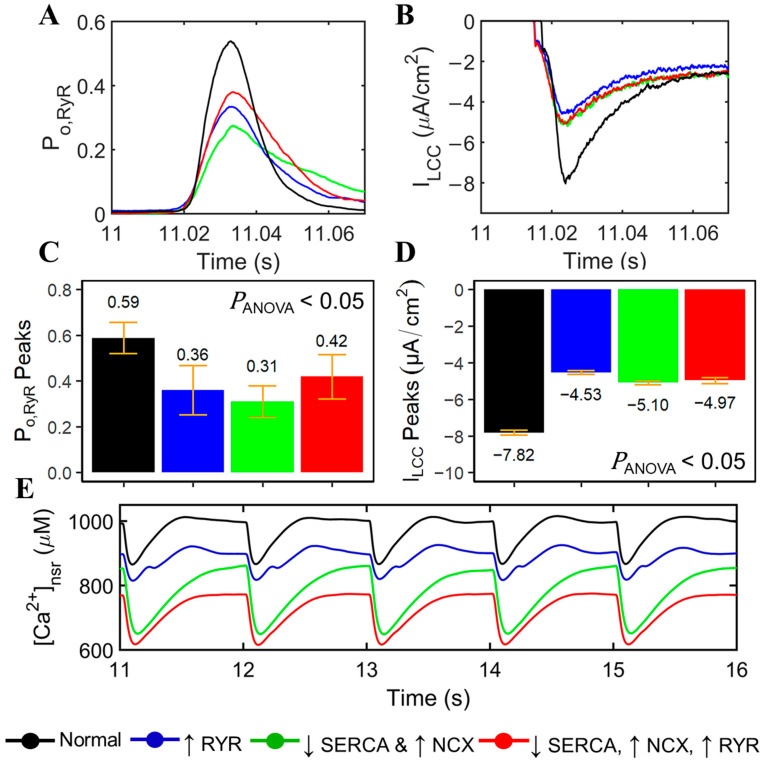
Comparisons of normal cardiac cell conditions vs. heart failure. To ensure steady state conditions, 20 s simulations at 1 Hz were performed. (**A**) Peak RyR2 open probability and (**B**) L-type calcium channel is generally decreased with each HF-related change, i.e., (1) RyR hyperphosphorylation (blue), (2) downregulated SERCA and increased NCX (green), and (3) combination of both (red) mechanisms. (**C**) On average, RyR2 open probabilities and (**D**) L-type calcium channel densities are significantly different from normal conditions in HF-related changes (P_ANOVA_ < 0.05). (**E**) Reductions in SR Ca^2+^ concentrations, both at systole and diastole, are observed in heart failure. In all cases except Normal, the K^+^ changes reflect those seen in HF. The legend on the bottom shows the increase or decrease of the different components.

In pathological conditions of failing hearts, substantial evidence suggests that cardiac contractility is associated with the reduction in SR Ca^2+^ at the cellular level [3,42,43,44]. However, little is known about its consequences for the functionality of the calcium-releasing units (CRUs). The next set of simulations in Figure 4 compares normal healthy myocytes to failing myocytes (red) compared to normal healthy myocyes (black). There are contributions for each of the changes to Ca^2+^ dynamics (increased RyR Ca^2+^ sensitivity, decreased SERCA expression, and increased NCX expression). In each of the simulations, the changes (except Normal) to the K^+^ current are included. RyR open probability and I_LCC_ density are decreased (Figure 4A and Figure 4B, respectively). Furthermore, because hyperphosphorylated RyR2s is a known physiological abnormality in HF, its activity is presumed to have direct anomalous behavior as part of the CRU. Therefore, peak amplitudes of RyR2 open probability and the I_LCC_ current were measured (Figure 4C and Figure 4D, respectively). We observe that the increased RyR sensitivity can decrease L-type calcium channel density (Figure 4D, blue bar) more than its own open fraction (Figure 4C, blue bar). A single-factor ANOVA test for independent measures also reveals that each of the HF-related changes of measured RyR and LCC amplitudes are significantly different (*p* < 0.05) from a normally functioning CRU. On the other hand, it is important to note that the overall Ca^2+^ levels in SR are decreased in HF. However, variation in levels of SR Ca^2+^ concentrations are consistent with each of the effects of hyperphosphorylated RyR2 versus impaired SERCA, and NCX (Figure 4E). It could also be inferred that the I_LCC_ peaks (Figure 4D) affect the allowable Ca^2+^ released by the SR during systole—the lowest peak of I_LCC_ by phosphorylated RyR alone also corresponds to the least SR flux into the cytosol (Figure 4E).

**Figure 5:**
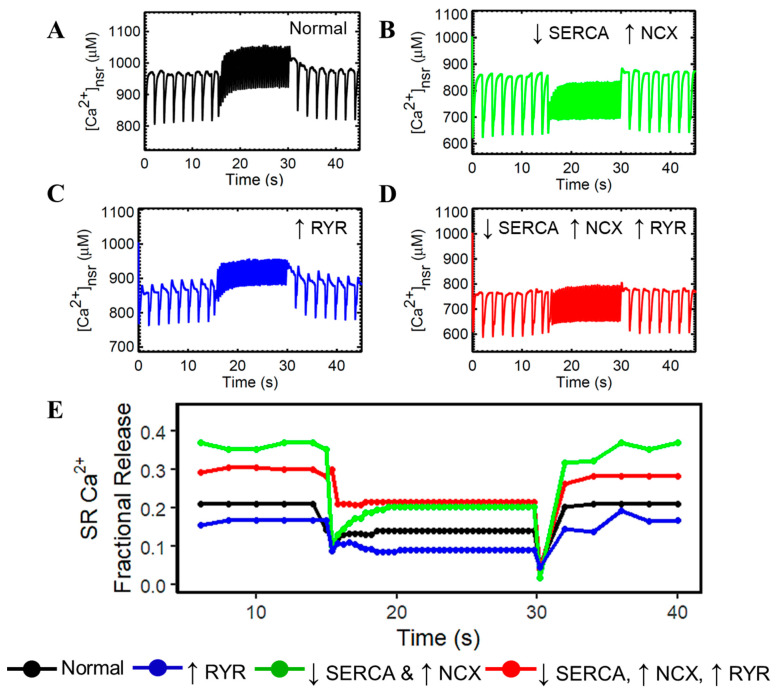
SR Ca^2+^ dynamic pacing (0.5–2.5–0.5 Hz) and fractional release. To ensure steady state conditions, 45 s simulations were performed. (**A**) Normal cardiomyocyte. (**B**) Due to impaired SERCA to re-sequester Ca^2+^ back into the SR and accompanied with stronger extrusion of Ca^2+^ by NCX, SR Ca^2+^ concentrations stay at 800 µM during 2.5 Hz pacing. (**C**) Increased RyR sensitivity shows diminished amplitudes between systole and diastole, but still exhibits a staircase effect. (**D**) the combined effects of three HF-related Ca^2+^ regulatory proteins display the absence of the Bowditch effect, which could be related to the deficient cardiac output seen in failing hearts. (**E**) SR Ca^2+^ fractional release is generally increased in cases of HF, but hyperphosphorylation of RyR2 alone accounts for lower Ca^2+^ release (blue). In all cases except Normal, the K^+^ changes reflect those seen in HF. The legend on the bottom shows the increase or decrease of the different components. The computed fractional release from the diastolic and systolic [Ca^2+^]_SR_ values from simulation results are in the Supplemental Materials: HF Supplementary Materials.rar.

In order to generalize this inference, we applied the same dynamic slow–rapid–slow pacing (0.5–2.5–0.5 Hz) in the network SR with the same isolated mechanisms (Figure 5): normal cardiomyocyte (Figure 5A), decreased SERCA activity and upregulated NCX (Figure 5B), hyperphosphorylated RyR2 (Figure 5C), combination of both cases (Figure 5D), and each of their respective SR Ca^2+^ fractional releases (Figure 5E). Once again, in all cases except Normal, the K^+^ changes reflect those seen in HF. On an important note, a diminished Bowditch or staircase phenomenon is a hallmark of heart failure [45]. This diminished Bowditch effect is observed in Figure 5B,D, largely caused by the downregulated SERCA and upregulated NCX.

### 3.4. Voltage Dependence and ECC Gain

**Figure 6:**
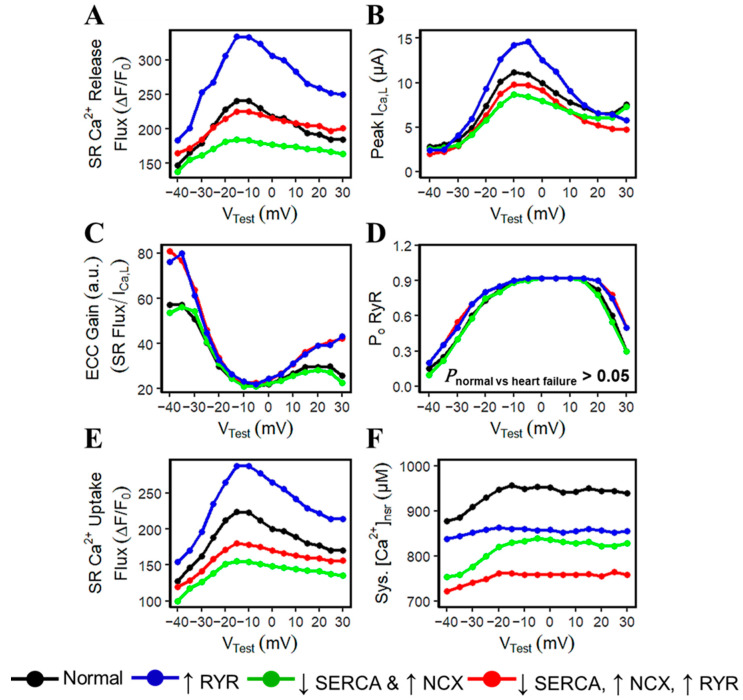
Excitation–contraction coupling gain. (**A**) The amount of SR Ca^2+^ release (in µM)—hyperphosphorylated RyR2s (blue) generally increase release flux as compared with impaired SERCA and upregulated NCX (green). (**B**) Peak I_CaL_ (in µA) observed—upregulation of I_NCX_ decreases the amount of Ca^2+^ current (green). However, as RyR2 is 50% more sensitive, more Ca^2+^ is released from the SR (blue). (**C**) ECC gain is altered at negative (−40 mV to −25 mV) and at positive potentials (from 15 mV and above). (**D**) RyR open probabilities (P_O,RyR_) are statistically insignificant and are also observed in experimental findings by Jiang et al. in failing canine and human hearts [7]. (**E**) A 50% reduction in SERCA activity accounts for the least Ca^2+^ sequestration into the SR. (**F**) Systolic SR Ca^2+^ levels are decreased in HF due to the decreased SR Ca^2+^ content and other relative HF conditions. The legend on the bottom shows the increase or decrease of the different components.

Gain is commonly referred to as a CICR amplification factor assessed through voltage dependence of affected currents and Ca^2+^ transients in the myoplasm [46]. Furthermore, it has been previously demonstrated experimentally that altered SR Ca^2+^ content can dramatically affect E-C coupling gain [47], and this phenomenon was presumed to be an effect of intra-SR Ca^2+^ on RyR2 gating [48]. Through a voltage-clamp protocol of 0.2 s depolarizing steps (V_Test_) from −40 to +30 mV, various SR Ca^2+^ fluxes and associated currents were obtained. Under voltage-clamp conditions, it shows that (1) hyperphosphorylated RyRs contribute more to the SR Ca^2+^ release flux than (2) upregulated I_NCX_ and decreased SERCA activity, and (3) all combined cases (i.e., hyperphosphorylated RyR, upregulated I_NCX_, and decreased SERCA) for HF (Figure 6A). It is important to note that the contraction of striated muscle is triggered by the opening of RyR Ca^2+^ release channels in the SR membrane in response to depolarization of the transverse invaginations of the plasma membrane by the T-tubules [49]. Hence, increased sensitivity of RyR2s in the heart muscle during the varying degrees of incremental increase in voltage is expected to release more Ca^2+^ from the SR.

On the other hand, E-C coupling in cardiac cells occurs by CICR where a small Ca^2+^ current (I_Ca,L_) through the L-type Ca^2+^ channels locally controls a larger Ca^2+^ release from the SR via RyR2s [50]. In the early studies of failing rabbit heart cells, I_Ca,L_’s current is decreased from −10 mV to positive membrane potentials due to the upregulation of I_NCX_ [51]. This phenomenon was observed in simulated HF; however, the I_Ca,L_ current can also increase in hyperphosphorylated RyR2s (Figure 6B). Data on I_Ca,L_ has been reviewed in detail in early studies of Hart [52] which described that I_Ca,L_ may be increased in mild hypertrophy, unchanged in moderate degrees of hypertrophy, and reduced in severe hypertrophy with failure [53]. Although the relationship between mechanisms of ECC gain and I_Ca,L_’s voltage dependence cannot be directly attributed to the cases of hypertrophied hearts, this could be an area of research worth pursuing.

The measurement of excitation–contraction coupling gain is computed as the ratio of peak SR Ca^2+^ release flux and the peak flux of [Ca^2+^]_i_ across the cell membrane [47,54,55]. This study aims to differentiate each HF’s underlying mechanism and the measurement of the EC coupling gain shows that increased RyR2 sensitivity corresponds to the highest gain in negative membrane potentials (Figure 6C). Changes in gain with other pathological and experimental conditions have been used to make inferences about the microscopic structures responsible for Ca^2+^ sparks [50], and more importantly, defective EC coupling cardiac hypertrophy and heart failure has been previously described [56] (relative characteristics of calcium sparks are discussed in the next section).

Hyperphosphorylation of RyR2s also minimally affects its open probability (Figure 6D). This statistically insignificant finding was previously reported by Jiang et al. [7] in the experimental recordings of failing canine and human hearts. To further support this finding, we conducted paired Student’s *t*-tests to mimic an experimental situation of a failing human cardiac cell from a single sample with different HF conditions, and all yielded to insignificant results (*p* > 0.05). This minimal effect to the RyR open fraction is because RyR activation is not sensitive to levels of Ca^2+^, but rather to their (a) proximity to the L-Type calcium channels [57], and (b) RyR2 adaptability when exposed to higher Ca^2+^ [58].

Moreover, because human cardiac cells rely on the SERCA pump to a lesser degree (~60%) than rodents (~90%), limited information is known in the direct implication of a reduction in SERCA activity in human failing hearts for overall contractile function [59]. This study demonstrates that the voltage dependence of SR Ca^2+^ reuptake by SERCA is directly related to its functionality, wherein the 50% reduction in SERCA activity in HF accounts for the least sequestration of Ca^2+^ back into the SR (Figure 6E, green line). And as previously described in Figure 4E, SR Ca^2+^ content is generally reduced in failing hearts and its systolic levels can vary with each underlying pathophysiological condition (Figure 6F).

### 3.5. Calcium Sparks in Failing Hearts

**Figure 7:**
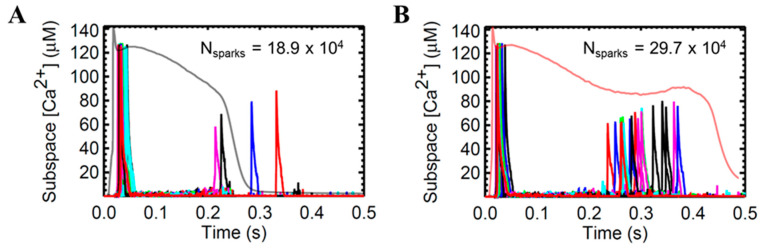
Representative sample (0.1% of 20,000 CRUs) of detected calcium sparks in the local subspace from 20 CRUs. Superimposed action potential from pacing at 0.5 Hz depicting relationship between AP and Ca^2+^ sparks (N_sparks_). (**A**) Ca^2+^ spark behavior in a normally functioning human AP. Minimal Ca^2+^ leak was observed in the late phase of the AP during extrusion of Ca^2+^ ions in cell repolarization. (**B**) Ca^2+^ spark behavior in heart failure with superimposed AP in the occurrence of early afterdepolarization (EAD). An increase in diastolic spark rate is evident in the late phase of the action potential, which causes APD prolongation and potential presence of EADs. The different colors in the plots indicate the Ca^2+^ spark from different CRUs.

Ca^2+^ sparks are discretized calcium release events due to random and collective openings of the RyR2 channels clustered in a calcium release unit (CRU). On a fundamental level, these sparks result from spontaneous openings of single SR calcium-release channels supported by ryanodine-dependent changes of spark kinetics [60]. The RyR is both the SR Ca^2+^ release channel and a scaffolding protein that localizes key regulatory proteins such as calmodulin, calsequestrin, and other proteins at the luminal SR surface [1]. Local CRU Ca^2+^ sparks were investigated using the mathematical model of Williams et al. [61] which includes spatial nanodomain determinants of individual CRU organization and a realistic number of 20,000 CRUs. The systolic [Ca^2+^]_i_ transient activates L-type Ca^2+^ channels at the surface membrane and at transverse tubules, which then elevates [Ca^2+^]_i_ locally in the dyadic subspace compartment between the t-tubular and terminal SR membranes. This situation is amplified when RyR2 clusters are activated by a locally elevated subspace [Ca^2+^]_i_ during CICR (see ref. [62] for a physiological review). This spontaneous local increase in intracellular calcium concentration from the SR produces calcium sparks (Figure 7A).

On the other hand, in early studies of cardiomyopathic ventricular cells, it was determined that failing ventricular cells were intrinsically predisposed to early afterdepolarizations and spontaneous depolarizations [63,64]. EADs have been linked to the pathogenesis of long Q-T syndrome and this reflects an increased propensity for abnormal repolarization and prolonged APD-related arrhythmias [63]. Computational studies suggest that the changes in heart failure, such as APD prolongation and an increase in late phase of Ca^2+^ release during AP, result in an increased diastolic spark rate [65] (Figure 7B). To illustrate this phenomenon, we paced the HF model in 0.5 Hz because the occurrence of EADs is at least 40% higher in lower pacing observed in failing cardiomyocytes [31] (Appendix A Figure A1).

**Figure 8:**
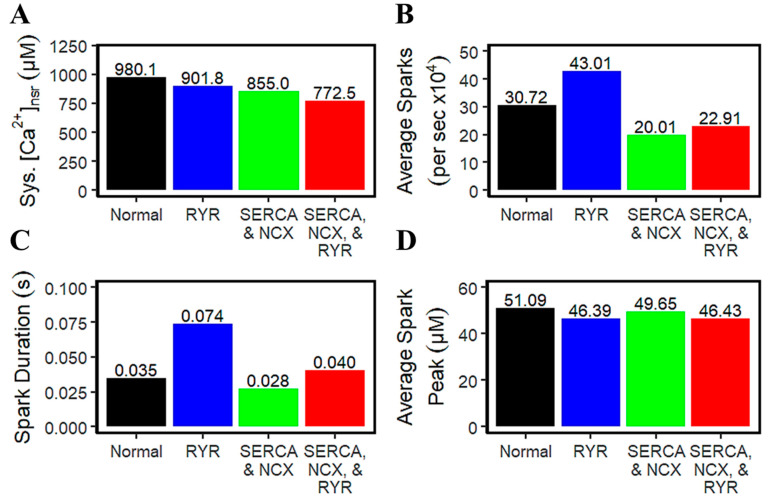
Comparisons of spark characteristics between normal and heart failure conditions. (**A**) Systolic SR Ca^2+^ levels are decreased in HF. (**B**) RyR hyperphosphorylation produces more sparks than normal and other HF conditions. (**C**) Similarly, increased RyR sensitivity prolongs spark duration. (**D**) Modest differences in average spark peaks in all stated underlying cardiac cell impairment.

**Figure A2:**
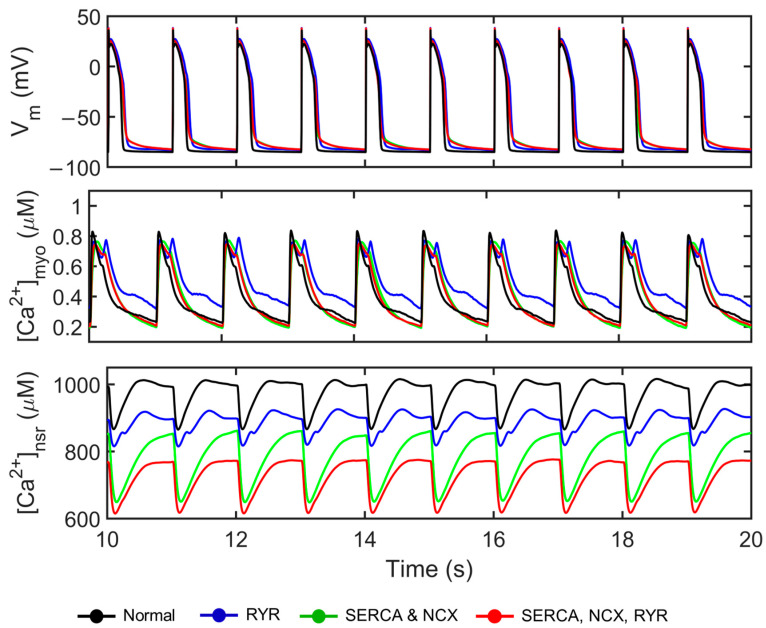
Simulation results of various HF conditions. RyR hyperphosphorylation (blue) displays more distinctive characteristics, especially in the prolonged calcium transient.

**Figure A3:**
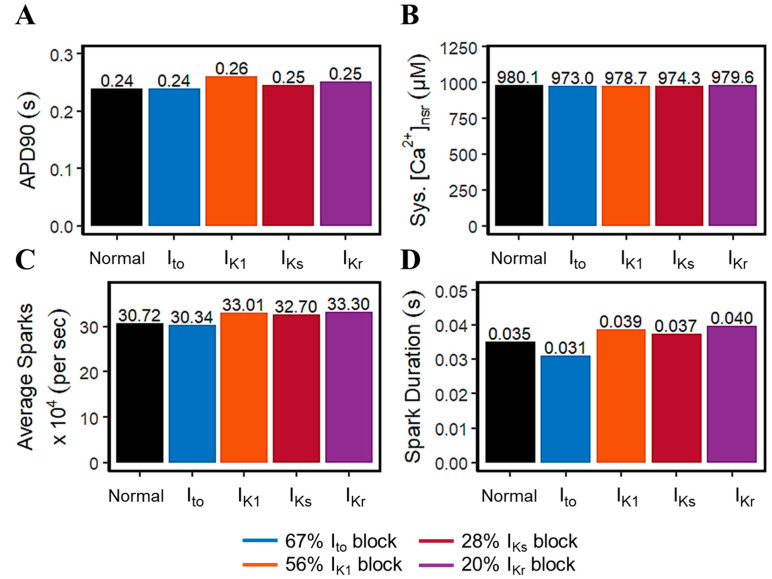
Comparisons of spark characteristics of normal versus each respective K^+^ block. (**A**–**D**) The differences in spark characteristics are negligible in all cases of downregulated repolarizing K^+^ currents.

Early investigations have emphasized that each RyR cluster is associated with multiple LCCs observed in both rat and rabbit ventricular cells [50,66,67], and results suggest that the small I_CaL_ could be a highly effective trigger of SR Ca^2+^ release. Accompanied during this SR Ca^2+^ release is the occurrence of calcium sparks which was discovered through the ryanodine-dependent changes of spark kinetics [60]. During systole in HF, SR Ca^2+^ levels are generally decreased (Figure 8A). However, differences in the average number of sparks seem to depend on the underlying pathophysiological condition in HF. Increased RyR2 sensitivity exhibits the most abundant number of sparks (Figure 8B) and displays a more lasting duration by two-fold longer than a normal cell (Figure 8C). This longer duration could also be related to prolonged Ca^2+^ transient (see Appendix A Figure A2 for simulation results). In contrast, there is little or no notable difference among all the HF mechanisms on each respective average spark peak (Figure 8D). On the same note, the variability of spark characteristics observed during K^+^ block conditions only (devoid of SERCA, INCX, and RyR dysfunction) are negligible (Appendix A Figure A3).

## 4. Discussion

In cardiac-modeling studies, the increase in spontaneous Ca^2+^ leak in failing hearts is simulated mainly through the increase in SR Ca^2+^ release by hyperphosphorylated RyR2, decreased outward potassium currents, and impaired calcium-extruding pumps such as SERCA and NCX. Accompanied with the reduced K^+^ currents, the cell then becomes oversaturated with Ca^2+^, which prolongs the APD, and the increase in cytosolic calcium is caused by the excessive calcium entry and reduced calcium efflux [68]. In contrast, decreased RyR2 mRNA expressions were also observed in failing human hearts [69,70,71] which helps explain the reduction of RyR2 open probability. Furthermore, in the context of calcium dynamics, it was observed by Arnáiz-Cot and colleagues [72] that the heart failure due to defective RyR2 led to a longer calcium transient decay, longer spark duration, and decreased spark peak, which prove to be consistent in this study.

### 4.1. SERCA and I_NCX_ vs. RyR: Alterations in SR Ca^2+^ Content

The presence of EADs at low pacing (0.5 Hz) is also possible throughout the oscillation of the membrane potential which re-depolarizes the myocyte due to the reduced outward currents. If spontaneous CICR from the SR occurs before repolarization is complete, the consequent stimulation of inward I_NCX_ may oppose and reverse repolarization [73]. In comparison, SERCA transports two Ca^2+^ ions per ATP consumed, whereas extrusion by NCX only pumps one Ca^2+^. The shift in competition from the SR Ca^2+^-ATPase toward NCX also tends to limit SR Ca^2+^ loading in heart failure [74], hence lowering SR Ca^2+^ content recovery. Moreover, it was previously observed in experiments using isolated RNA human left ventricular myocardium of 30 cardiac transplant recipients with end-stage heart failure [75] that the overall expression of SERCA mRNA was diminished by 50%, thus altering the Ca^2+^ handling that contributes to systolic and diastolic dysfunction in heart failure patients.

During heart failure, RyR2s are hyperphosphorylated, which increases their sensitivity to Ca^2+^. In the model, this is simulated by increasing the Ca^2+^ sensitive RyR opening rate, thereby increasing open probability for a given subspace and SR Ca^2+^ level. However, with the increased RyR open probability, there is more Ca^2+^ leak out of the SR, reducing the SR load as shown by this model (Figure 2E black vs. blue traces and in previous modeling and experimental work) [2,5,6,61,76]. A decrease in SR Ca^2+^ load can lead to a decreased probability of sparks. In the simulations, there is a reduction in the peak RyR open probability, but an increase in the RyR open probability (and number of Ca^2+^ sparks) late in the action potential and during diastole. Hence, the total number of Ca^2+^ sparks and non-spark leak increases in spite of a reduction in peak RyR open probability during the AP.

Previously reported evidence suggests that cardiac contraction would be further reduced if it were not for concomitant modulations to RyR2s, which sensitize release clusters to release Ca^2+^ [77]. These SR Ca^2+^ alterations were observed and reported to increase Ca^2+^ spark frequency and fractional SR Ca^2+^ release during ECC in failing cardiomyocytes as compared to healthy cells [78,79,80]. On the other hand, Kubalova et al. reported that an event of diminished SR Ca^2+^ release in failing myocardium could be explained by the increased sensitivity of RyR2s [80]. From Figure 5B, this study shows that SR Ca^2+^ fractional release could be altered depending on the impairment of calcium-related proteins—(a) diminished SERCA function and enhanced I_NCX_ can increase SR fractional release while (b) increased sensitivity of RyR2s can decrease it. Determining these phenomena could provide a therapeutic advantage for drug targets.

EC coupling gain was clearly altered at negative membrane potentials by changes in SR Ca^2+^ content and fluxes. Other studies have also reported altered E-C coupling gain independent of SR Ca^2+^ load, although this was not considered a universal finding [81]; pirfenidone treatment for human pulmonary fibrosis increases I_LCC_ expression by twofold and was shown to increase ECC gain at negative potentials [82]. This study suggests that ECC gain can also be increased by hyperphosphorylation of RyR2s as suggested by the results of voltage-clamp experiments. As pointed out by Trafford et al. [83], sudden increase or decrease in Ca^2+^ release through the RyR2 for a given stimulus will cause alterations in the SR Ca^2+^ load. The increase in L-type Ca^2+^ current also increases the Ca^2+^ entry into the cell, with a resulting higher SR Ca^2+^ content which was also observed experimentally in rat and ferret ventricular myocytes [84]. In the context of Timothy syndrome (LQT type 8), a disease caused by a gain-of-function mutation in the L-type calcium channels [85], Ca^2+^ influx is increased, which also prolongs the APD that causes arrhythmogenic effects. It was demonstrated by Drum et al. [86] in LQT-8 mouse ventricular cells that even small alterations to the LCCs were sufficient to induce maximal changes in resting systolic and diastolic intracellular calcium, which eventually resulted into an increased SR Ca^2+^ load and increased ECC gain.

### 4.2. Importance of Transient Outward Potassium Current (I_to_) Effects

AP morphology and duration are directly influenced by the inward and outward currents, and these current abnormalities have been comprehensively described in human heart-failure experiments [38,53]. Several studies examining I_to_ in heart failure used calcium-buffered internal solutions, which eliminates any possible role of calcium-dependent processes [38] and multiple pieces of evidence suggest that I_to_ can also influence overall action potential duration [28,63,87]. Moreover, it was observed that I_to_ downregulation in failing left and right ventricular human cardiac cells accounts for the diminished phase 1 repolarization of the action potential [20,88] and multiple diseases stemming from this altered I_to_ function have also been reported [89,90]. The cause of Ito changes has been linked to changes in transcription factor activation [91]. The model simulations show that with the reduction in I_to_ the notch in the AP disappears, leading to increased membrane potential when the peak L-type Ca^2+^ occurs. With the increased membrane potential, there is less driving force for Ca^2+^, thereby reducing the peak L-type Ca^2+^ current. This leads to less triggered RyR activity, further impacting Ca^2+^ dynamics. This research also demonstrates that I_to_ downregulation, other than the expected loss of “notch” in the action potential, can also lead to slower time-to-peak transfer of calcium to the cytoplasm. This kinetic trait was experimentally recorded and observed as a slowed Ca^2+^ transient under the conditions of hypertrophy or heart failure [38,39].

### 4.3. Limitations

#### 4.3.1. Rapid Delayed Rectifier Potassium Current (I_Kr_) Downregulation

The downregulation of I_Kr_ in failing human cardiac cells is currently being debated and its experimental evidence is still undergoing investigation. Its protein expressions from animal heart-failure models were reported to be inconsistent: felines with hypertrophy and rabbits on pacing-induced HF were observed to have significantly lower I_Kr_ expression than normal left ventricular cells [92,93], while, on the other hand, rats, guinea-pigs, and canines show no changes [31,94,95]. Recently, Holzem et al. [22] observed that in failing human left ventricular cell, they found no statistically significant differences in the mRNA expression levels when comparing failing to donor human heart tissue. However, they did emphasize in their experiments that I_Kr_ is functionally downregulated in human heart failure—the hERG1a mature protein had a 0.55-fold reduction (*p* = 0.51) in the epicardium of failing compared with healthy hearts and total hERG1a protein trended toward reduction. Moreover, the stoichiometry of hERG1a:hERG1b isoforms was altered in failing versus donor hearts, with 0.58-fold reduction in the epicardium. Another perspective involves cardiac safety—the analysis of I_Kr_ blocking effects in associative risk of drug-induced arrhythmia is mandatory in the development of new drugs [21,96]. Therefore, we slightly decreased the conductance of I_Kr_ in this study to emulate a modest contribution to APD prolongation as seen in experimental findings.

#### 4.3.2. Other Ionic Currents Present in Heart Failure

Several alterations seen in ionic current behaviors and Ca^2+^ regulatory proteins were previously described in multiple cases of hypertrophy and heart failure. For example, a mutation in the *CACNA1C* gene encoding the L-type calcium channel (I_LCC_) was associated with reduced voltage-dependent inactivation and displayed a propensity for arrhythmogenesis [97]. Another example is that the functional changes, especially in the gain-of-function, of the late sodium current (I_Na_) inhibition were considered a major contributor to electrophysiological and calcium dynamic abnormalities from patients with human hypertrophic cardiomyopathy (HCM) [98]. However, this study focused on the contributions of the K^+^ currents and other calcium regulatory proteins such as SERCA, RyR, and the sodium–calcium exchanger (I_NCX_) due to their relative contribution to repolarization and prolonged APD.

It was previously described by Tomaselli et al. [38] that there is a dispersion of action potential duration in the ventricle of both man [99] and animals [100] and it is possible that changes in the expression of K^+^ currents could enhance this dispersion of action potential repolarization. In the ventricular action potential, I_Ks_ is a major contributor to the ventricular “repolarization reserve” by providing a redundant current to maintain rapid repolarization during increases in cardiac rate or during conditions in which other ventricular potassium currents are reduced, specifically the I_Kr_ current in humans [101]. From a spatial perspective, Q-T interval dispersion accompanied in sudden expected death in chronic heart failure has been reported [102]. Temporally, the beat-to-beat Q-T interval variability was also evidently described in ischemic and non-ischemic dilated cardiomyopathy, which reflects withdrawal of parasympathetic tone accompanying heart failure [103]. Potassium currents are responsible for the repolarization of the cardiac action potential, and spatial and temporal dispersion of repolarization are altered in the failing human heart. This dispersion of repolarization has a major role in long Q-T, short Q-T, and Brugada syndromes and may also underlie polymorphic ventricular tachycardia and ventricular fibrillation [38,104,105,106].

#### 4.3.3. T-Tubule-Remodeling During Heart Failure

In HF cardiomyocytes, the rising phase of the Ca^2+^ transient is slower than healthy cells which contributes to its impaired contractility. It was previously reported by Louch et al. that dyssynchronous Ca^2+^ transients in failing mouse ventricular myocytes results in a subset of Ca^2+^ sparks with slow kinetics and they suggest that this observation is predicted to be the result of the reorganization of the calcium release units [107]. They also report that these slowed sparks occur at intact failing cardiac dyads and not in orphaned RyR2s. In mice, a re-organization of the t-tubular systems and orphaning of the RyRs (decoupling form the L-type Ca^2+^ channels in the dyad) observed by Wagner and co-workers was shown to provide a local mechanism for delayed subcellular Ca^2+^ release and action potential prolongation through computational modeling [17]. However, although in human heart ventricular myocytes the t-tubular system is not as dense as in rodents, there are still changes seen in heart failure [108]. In dog and pig hearts, which have a more similar AP to human than rodents, there is a large number of areas of the healthy ventricular myocyte showing low t-tubule density. However, similar studies in healthy human hearts are scarce [108,109]. In one such study, Lyu and colleagues observed t-tubule remodeling and RyR orphaning in aging otherwise healthy human hearts [110]. On the other hand, a recent study by Seidel and co-workers used samples from end-stage HF patients undergoing left ventricular assist device surgery [111]. The patients were stratified into two groups, one that had ejection fraction improvement before surgery and one that did not. The group showing no improvement after surgery had decreased t-tubule density and RyR orphaning compared to the group that showed ejection fraction improvement after surgery. In the group with t-tubule remodeling, there the Ca^2+^ transient was delayed and more asynchronous, similar to the modeling predictions of Wagner and co-workers in rats [17]. In fact, Wei and co-workers have suggested that t-tubule remodeling is crucial for the transition from ventricular hypertrophy to heart failure [112]. In Figure 8, this study also demonstrates that the slowed kinetics and higher occurrences of calcium sparks in failing hearts are largely influenced by the altered RyR2 function through increased sensitivity. Relatedly, however, potassium current blockade as described in Figure 2 minimally contributes to the overall spark characteristics of failing hearts. Therefore, this insight prompts the importance of investigating abnormal CRU reorganization at a cellular perspective in future studies.

## 5. Conclusions

Diseased or failing hearts are known to pathologically regulate a wide variety of ion channels and Ca^2+^-handling proteins, which eventually contribute to their associated ventricular arrhythmogenesis. Alterations in contraction and relaxation of failing hearts can be directly associated with corresponding alterations in Ca^2+^ cycling. Specifically, this study demonstrates that SERCA, NCX, and RyR2 dysfunction, along with the downregulation of the cardiomyocyte’s potassium currents, could substantially contribute to heart failure. Despite major progress in cardiovascular studies, heart failure still remains one of the leading causes of death. Therefore, early detection of defective cardiac cell behavior is crucial for preemptive onsets of abnormal cardiac contractility and overall help mitigate this life-threatening disease.

## Figures and Tables

**Table 1:** Normal and heart-failure simulation settings.

Parameter	Normal	Heart Failure	Reference
I_K1_ Inward rectifier potassium current	G_K1_ = 0.238 mS/µF	Reduced 56%	[20,28]
I_to_ Transient outward potassium current	G_to_ = 0.618 mS/µF	Reduced 67%	[20,28]
I_Kr_ Rapid delayed rectifier potassium current	G_Kr_ = 0.0488 mS/µF	Reduced 20%	[22,29]
I_Ks_ Slow delayed rectifier potassium current	G_Ks_ = 0.0224 mS/µF	Reduced 28%	[20]
RyR2 sensitivity	$k_{ryr}^{+}$ = 14 (unitless)	Increased 50%	[9,10,27]
Ap SERCA pump concentration	150 µM	Reduced 50%	[19,30]
I_NCX_ Sodium–calcium exchanger	G_NCX_ = 1000 µA/µF	Increased 100%	[23]

## Data Availability

Model code is available in the Supplemental Material. The authors should be contacted for any other data or help with the code.

## Supplementary Materials

The following supporting information can be downloaded at: https://www.mdpi.com/article/10.3390/biom14111371/s1, HF Supplementary Materials.rar.
